# “I Just Can’t Do It Anymore” Patterns of Physical Activity and Cardiac Rehabilitation in African Americans with Heart Failure: A Mixed Method Study

**DOI:** 10.3390/healthcare3040973

**Published:** 2015-10-15

**Authors:** Margaret McCarthy, Stuart D. Katz, Judith Schipper, Victoria Vaughan Dickson

**Affiliations:** 1 New York University College of Nursing, New York, NY 10010, USA; E-Mail: vdickson@nyu.edu; 2 New York University Langone Medical Center, New York, NY 10016, USA; E-Mails: stuart.katz@nyumc.org (S.D.K.); judith.schipper@nyumc.org (J.S.)

**Keywords:** heart failure, exercise, cardiac rehabilitation, minorities

## Abstract

Physical activity and cardiac rehabilitation (CR) are components of heart failure (HF) self-care. The aims of this study were to describe patterns of physical activity in African Americans (*n* = 30) with HF and to explore experience in CR. This was a mixed method, concurrent nested, predominantly qualitative study. Qualitative data were collected via interviews exploring typical physical activity, and CR experience. It was augmented by quantitative data measuring HF severity, self-care, functional capacity and depressive symptoms. Mean age was 60 ± 15 years; 65% were New York Heart Association (NYHA) class III HF. Forty-three percent reported that they did less than 30 min of exercise in the past week; 23% were told “nothing” about exercise by their provider, and 53% were told to do “minimal exercise”. A measure of functional capacity indicated the ability to do moderate activity. Two related themes stemmed from the narratives describing current physical activity: “given up” and “still trying”. Six participants recalled referral to CR with one person participating. There was high concordance between qualitative and quantitative data, and evidence that depression may play a role in low levels of physical activity. Findings highlight the need for strategies to increase adherence to current physical activity guidelines in this older minority population with HF.

## 1. Introduction

Heart Failure (HF) affects over 5.7 million adults in the United States with Black men and women having the highest prevalence [1]. African Americans had the highest risk of developing HF and have the highest proportion of HF that is not preceded by myocardial infarction [1].This risk differential reflects disparities in the prevalence of hypertension and diabetes, as well as the effects of disparate socioeconomic status on access to medical care. Less than 25% of African Americans with HF receive treatment according to recent guidelines, and have a higher fatality rate than Whites [2]. Additionally, in 2012, there were 870,000 new cases of HF in adults >55 years old [1].

Exercise is recommended as one of many self-care behaviors for those with HF, but adherence rates are low [3,4]. In a study of 139 patients with HF, over half stated they engaged in no regular physical activity [5]. In the United States, very few non-Hispanic Black adults (17.3%) meets physical activity guidelines for aerobic and muscle-strengthening activity [6], which is unfortunate given insufficient physical activity accounts for almost 12% of the risk of myocardial infarction, even after accounting for other cardiovascular risk factors [7]. Racial disparities in cardiac rehabilitation participation (CR) have been previously reported. In a review of Medicare beneficiaries after myocardial infarction or coronary artery bypass graft surgery, Whites (19.6%) were more likely than non-Whites (7.8%) to participate in a CR program [8]. Additionally, patients with low socioeconomic status report greater barriers to CR, including lack of referral, and lower enrollment and participation rates than those of higher socioeconomic status [9]. Therefore, the aims of this mixed methods study were to describe the patterns of physical activity in a small sample of low-income African Americans with heart failure, and to explore the pattern of referral and participation in CR.

Engagement in physical activity including cardiac rehabilitation is influenced by multiple factors. An integrative review on the barriers to CR participation included referral, comorbidities, transportation, and knowledge, as barriers. Racial disparities were also found in the referral process, with minority women less likely to receive a referral [10].

However, most of the research describing physical activity and exercise practices in patients with HF has been limited to Caucasian populations [11,12] or the race/ethnicity has not been disclosed. There is a need to know more about the populations that are seen in daily clinical practice. This is challenging since it includes those groups not typically studied in clinical trials: women, elderly and minority groups [13]. More recently, a large clinical trial of exercise in HF with over 2000 subjects included 40% racial and ethnic minority adults [14].

It has previously been reported that engagement in self-care in an ethnic minority population was behavior-specific, with adequate adherence to medication regimens but poor adherence to other self-care behaviors [15]. In that sample, subjects described poor adherence to symptom monitoring, which is essential for engaging in a program of physical activity. Cultural beliefs including the meaning of HF and its inevitability as a diagnosis, along with social norms, seemed to influence engagement in self-care in the ethnic minority population. Therefore, the aims of this study were to analyze qualitative and quantitative data collected in a mixed methods study to describe the physical activity patterns of African American patients with HF and examine patterns of CR referral and participation.

### Theoretical Framework

The Situation-Specific Theory of Heart Failure Self-Care [16] guided this study. Two components comprise the Self-care of HF model: self-care maintenance, composed of symptom monitoring and treatment adherence, and self-care management, where a patient recognizes a change in health, decides to take action and evaluates the effectiveness of the treatment. Integral to this theory is confidence, which is thought to moderate and/or mediate the effect of self-care on health outcomes [16]. Routine physical activity can be incorporated into this model as one of the recommended self-care behaviors that are part of a patient’s daily self-care.

## 2. Methods

This secondary analysis was part of a larger study examining the sociocultural influences on HF self-care in a sample of African Americans [15]. This was a mixed method, concurrent nested study with the quantitative data embedded in a predominantly qualitative study. Given the exploratory aims of this study, the priority was qualitative data, [17] which were collected via semi-structured interview exploring self-care practices, typical physical activity, and questions about CR referral and participation. The quantitative data were used to describe the sample and augment qualitative data. Physical activity and functional status data were collected with valid and reliable instruments. The study received the appropriate Institutional Review Board (IRB) approval of New York University School of Medicine, and the Health and Hospital Consortium in 2009.

### 2.1. Sample and Setting

This was a convenience sample of patients attending an urban HF clinic in a large municipal hospital that provides outpatient care (including medications) at little or no cost. This clinic serves a low socio-economic population. A research assistant, who was not involved in clinical care, regularly attended the heart failure clinic and distributed IRB approved flyers to potential participants in the waiting area. Patients that self-identified as African American were invited to participate in the study. Other inclusion criteria included: confirmed HF diagnosis based on echocardiography or clinical evidence for at least three months; relatively stable New York Heart Association Class III or IV; and over the age of 18 years. The diagnosis of HF could include both reduced or preserved ejection fraction, as well as ischemicor non-ischemic. Those unable or unwilling to provide informed consent, or with a history of a prior neurological events that could cause dementia, or those unable to perform tests or participate in an interview were excluded.

### 2.2. Data Collection and Analysis

The research assistant collected both qualitative and quantitative data in one session in the HF clinic. After obtaining informed consent, data collection sessions started with the administration of quantitative instruments. Then, the research assistant, trained in qualitative interviewing, conducted the qualitative interview. Each data collection session lasted approximately one hour. Participants were given a small non-coercive incentive to compensate for their time.

### 2.3. Quantitative Data

Valid and reliable instruments were used to collect socio-demographic data, physical activity information, self-care, physical functioning and depression. Information regarding recent physical activity was obtained from the question “During the past week (even if it was not a typical week), how much total time did you spend on exercise (including strengthening exercises, walking, swimming, gardening, active housework or other types of aerobic exercise)?” The responses ranged from “none” to “more than three hours per week”. Although exercise is often referred to as a division of physical activity that is more structured [18], in this study the terms are used interchangeably. Participants were also asked “What were you told about exercise by your health care provider?” with the following possible responses: “not to exercise”; minimal exercise only”; use a home or out-of-hospital program”; “attend cardiac rehabilitation”; or “nothing”.

Self-care including exercise was measured by the Self-care of Heart Failure Index (SCHFI) version 6.2. The SCHFI has three scales measuring self-care maintenance, management and confidence and a score of ≥70 indicating adequate self-care [19]. There is an exercise question “How routinely do you exercise for 30 min?” embedded in the self-care maintenance scale. The Chronbach’s alpha for each subscale were mixed (SCHFI maintenance = 0.665; SCHFI management = 0.500; SCHFI confidence = 0.827). The SCHFI management scale was lower than desired and may reflect some of the inconsistencies seen in this population.

The Duke Activity Status Index (DASI) [20] is a 12-item questionnaire measuring functional capacity with a possible score ranging from 0 to 58.2; in this study it had an alpha level of 0.77. The Patient Health Questionnaire (PHQ-9) [21] was used to measure depression (score of ≥10 indicating depressive symptoms). The alpha level for this study was 0.787.

New York Heart Association (NYHA) classification was measured using a standardized survey [22] and used to describe the sample. This classification ranges from NYHA class I (no limitation of physical activity) to class IV (symptoms of HF present at rest) [23]. A single question on the sociodemographic questionnaire assessed quality of life (“Overall, how would you rate your quality of life? “poor” “satisfactory” “good” “very good”). The quantitative data were analyzed using SPSS (version 18). Descriptive statistics of the sample, and correlations of PHQ-9, DASI and self-care maintenance were computed.

### 2.4. Qualitative Data

Narratives about physical activity or exercise as a component of HF self-care were elicited using a semi-structured interview guide. Each interview was tape recorded and transcribed verbatim. Each qualitative interview began by asking two open-ended questions (“Tell me about your heart failure”. To gain insight into physical activity, participants were asked about typical daily activities (“Tell me about a typical day for you”), followed by more specific questions about exercise. Finally, they were asked about experience with cardiac rehabilitation referral (“What have you been told about cardiac rehabilitation?”) and experience if individuals indicated if they had attended. The qualitative data were analyzed with thematic content analysis [17], using Atlas.ti (V6). This method involves identifying codes and themes within each case and then looking for commonalities that may transcend cases. Two researchers, who were not involved in clinical care, conducted each stage of this analysis. Victoria Vaughan Dickson is an expert in qualitative data analysis and Margaret McCarthy received training in qualitative analysis. In this study, this entailed a preliminary line-by-line review of the transcriptions that yielded clusters of data labeled into brief headings of physical activity (for example “past physical activity” “current physical activity”). Themes derived from these data revealed patterns of physical activity and cardiac referral experiences. Finally, emerging themes within-cases were compared across cases to identify commonalities. Methodological rigor was maintained through an audit trail and periodic peer debriefing with experts in HF and minority population research that supported the credibility of the study [24].

### 2.5. Data Integration

In the final step of analysis, the data were integrated through assessment of concordance or agreement between quantitative data (how much exercise was completed in past week) and qualitative descriptions of typical daily activity. The percent of agreement between the two sources was calculated. Given the degree of depressive symptoms evidenced in the PHQ-9, the qualitative data were then reviewed for evidence of depressive symptoms affecting physical activity. An informational matrix [25] was developed to compare and contrast the emergent qualitative themes and the quantitative evidence of recent physical activity across the cases.

## 3. Results

### 3.1. Sample Characteristics

This was a sample of 30 participants who self-identified as African American but were born in many different countries including the U.S., the Caribbean and Africa. The mean age was 60 ± 15 years, 60% were men, with a mean BMI in the overweight category (29.3 kg/m^2^). The majority (65%) was NYHA class III HF with a mean of 10.9 ± 4.7 years of education. Most (60%) were single, divorced or widowed with the majority (83%) having some type of government insurance (Table 1).

**Table 1:** Clinical characteristics of the study participants (*n* = 30).

Variable	
Male, *n* (%)	18 (60)
Age, years (mean ± SD)	60 ± 15.2
Education, years (mean ± SD)	10.9 ± 4.7
Marital Status	
Married or Co-Habitate, *n* (%)	12 (40)
Single, Divorced, or Widowed, *n* (%)	18 (60)
NYHA	
Class III, *n* (%)	20 (65)
Class II, *n* (%)	10 (35)
Body Mass Index kg/m^2^ (mean ± SD)	29.3 ± 6.3
Government Insurance, *n* (%)	25 (83)

*Note*: NYHA = New York Heart Association; SD = standard deviation; kg/m^2^ = kilogram per meter squared.

### 3.2. Quantitative Results

Almost half of the sample (43%) reported that they did “none”, or “less than 30 min of exercise in the past week”. When asked what they were told about exercise by their health care provider, almost one in four (23%) were told “nothing” about exercise, and over half (53%) were told to do “minimal exercise only”.

The mean DASI score was 16.8, which translates into approximately five metabolic equivalents (METS). This corresponds to an ability to perform moderate activity, such as walking at a leisurely pace. The DASI scores ranged from the lowest possible score of 0 (2.7 METS) to the highest of 58.2 (10 METS). The mean PHQ-9 score was 7.6 ± 5.3, but 40% of the participants had scores of 10 or greater indicating depressive symptoms. The PHQ-9 score was significantly correlated with the DASI score. Those with higher levels of depression had lower levels of functioning on the DASI (*r* = −0.318; *p* = 0.024).

The mean score for each subscale of the SCHFI was less than adequate (maintenance = 60 ± 18; management = 51 ± 18; and confidence = 62 ± 18). Fewer than 25% achieved an adequate score of ≥70 on any scale. The participants’ quality of life tended to be rather low, with only 11% describing their quality of life as very good, and 21% stating it was poor.

### 3.3. Qualitative Results

The narratives about exercise and physical activity revealed insight into the patterns of physical activity and the impact on daily life among this ethnic minority population. Specifically, reflections of past physical activity uncovered a theme of intrinsic benefits that included enjoyment of physical activity. Two related themes stemmed from the narratives describing current physical activity: “given up” and “still trying”.

### 3.4. Past Physical Activity

Individuals in this study spoke about the activities that they used to enjoy but can no longer do (“*…I was really athletic at one time … I miss … playing basketball…*”). Importantly, they discussed how HF symptoms interfered with their daily physical activity and the consequential impact on their quality of life. A female with NYHA class III HF, recounted her active lifestyle before HF symptoms restricted both physical and social activities and socialization… “*I used to walk a lot*, *I used to be happy go lucky going places and now … it’s like I feel like I’m tied down*. *I can’t really do the things I like to do because I end up getting sick again … I used to go out dancing*”.

### 3.5. Current Physical Activity

The participants spoke about what they were able to do now that they had HF, usually as measured against previous physical activity. A 70-year old male recalled “*I can’t do things anymore*. *I have to take it easy*. *I cannot run*, *play baseball like I used to*, *with my grandkids*” One 65 year old male said simply “*I am not exercising the way I was*”.

Fear emerged as a factor that influenced one’s willingness to engage in physical activity now. For example, fear that physical activity would precipitate symptoms affected the current daily activities of one 70-year-old female with Class III HF. “*…I’m afraid if I go out*, *I can’t make it back … I can’t even carry three pounds of nothing*, *a carton of milk is too much*. *That’s very hard*”.

### 3.6. “Given up”

Generally, the narratives in this sample revealed significantly limited physical activity levels that impacted all aspects of daily life and as a result many described having “Given up”. For example, a 63-year-old male with Class III HF described a typical day as “*…Sometimes I don’t feel good and I just stay in bed all the time*. *I just get up*, *eat something and go back to sleep … all day … sometimes for three days*”.

Another 72-year-old woman with Class III HF talked about barely being able to let her home health aide in the door in the morning. “*…now I don’t even bother to get up*. *Sometimes I can’t get up … and let my girl in*. *Sometimes I go back to bed ‘cause that’s all I have any energy for*”.

Individuals described “giving up” despite the desire to remain active. “*I don’t really do nothing now. I just do things for myself, just for me … I want to go do things all the time. I never lost interest. But I just cant’ do it no more*”.

### 3.7. “Still Trying”

However, despite symptoms of HF, some remained optimistic. One 70-year-old man with NYHA class III HF expressed his belief that if he could just get back to the gym he would be all right. “*If I go back to the gym … I think I will be right where I was before*”.

A 60-year-old female with NYHA class III HF recognized the importance of exercise, but adjusted her actively level given HF symptoms. “*I still go to the beach but very little swimming ‘cause I find when I swim I get tired quick so I have to still be careful*. *So I have to cut down on a lotta activities … but I still try…*”

### 3.8. Cardiac Rehabilitation Referral and Experience

When asked about cardiac rehabilitation, six participants (20%) stated they were referred to cardiac rehabilitation. No participants recalled asking for information about exercise. Only one person completed a program. He described attending the exercise program twice weekly, and its positive impact on his health “*I think it really helped me with the diabetes*”. There were three reasons that individuals cited for not attending CR: lack of knowledge, no insurance coverage, and inability to complete the exercise. Twenty-four participants reported they had never been referred; many knew nothing about CR. A typical response was, “*No*. *They never told me nothing about that*”.

### 3.9. Data Integration

Qualitative descriptions of a typical day were triangulated with quantitative responses regarding how much exercise was completed in the past week (categorical responses ranging from “none” to “greater than 3 h”). There was 82% concordance, or agreement, between qualitative descriptions and the quantitative data. Some that were not concordant appeared to overestimate the amount of exercise done in the past week, while qualitative descriptions of their typical day revealed very little activity at all. Reasons for decreased physical activity centered on a low level of physical functioning and conditioning, which is consistent with the majority of our sample having NYHA Class III HF. However, there was also compelling evidence that depression may play a role in low levels of physical activity.

A 57-year-old woman with evidence of depression (PHQ-9 = 11) and a low DASI score (1.75) indicating low physical function, reported less than 30 min of exercise in the past week but in the past had enjoyed dancing. She described trying to dance recently at a family party but had to sit down after less than five minutes “*I used to go out dancing*. *But now I can’t*”.

Although the mean DASI score reflected an ability to do moderate activity, the qualitative descriptions of their day reflected much less; and revealed that depression might be a factor. For example, one man revealed how HF symptoms and depression have affected him, “*after you get chest pains and everything*, *you*, *you don’t feel like doing nothing*, *you just get depressed*”.

## 4. Discussion

This mixed method study examined the patterns of physical activity and cardiac rehabilitation referral in a sample of older African American adults with HF, and revealed insights into influences on their daily physical activity. Specifically, low levels of physical activity and elevated levels of depressive symptoms were found in this population. Our study provides important information about why physical activity may be so poor, including the prevalence of symptoms that interfere with both routine and planned physical activity, as well as a lack of information about how to engage in regular physical activity or exercise.

Unfortunately, in general, the self-care practices in this sample were sub-optimal, which parallels their lack of physical activity as a part of self-care, and is not a new finding in low-income or ethnic minority populations [15,26,27] The principal signs of HF (dyspnea and fatigue) [4] which results in exercise intolerance, were seen in this sample. Participants reported symptoms of extreme fatigue and shortness of breath, which interfered with day-to-day physical activity. Although regular exercise can improve these primary symptoms of HF [28], the participants in our study exercised very little according to both the quantitative data and qualitative accounts. Our study provides important insight into this paradox. Despite current clinical guidelines, our sample reported that they received minimal explicit instruction on how to incorporate physical activity into their daily self-care routine. The factors that contribute to this finding are likely complex and cannot be discerned from the current study design. One of the goals of Healthy People 2020 is to increase the proportion of medical office visits that include counseling or education about exercise with patients diagnosed with heart disease, diabetes or hyperlipidemia [6]. This inconsistency is particularly critical for ethnic minority populations who have a higher prevalence of HF, lower levels of physical activity, and may benefit from direct and explicit counseling during an office visit. Additionally, higher levels of physical activity have been associated with better cognitive function in older adults with HF [29]. This may be a much-needed additional benefit in this population of adults with HF. The results of a systematic review of cognitive impairment in HF reveal adults with HF have increased odds (OR = 1.62; 95% CI: 1.48–1.79; *p* < 0.0001) of cognitive impairment [30].

In addition, few individuals in our sample reported they had received a referral to cardiac rehabilitation. According to the American Heart Association [3] a tailored exercise program is viewed as a safe, adjunctive component of treatment for HF patients. In addition to providing a place for monitored exercise, cardiac rehabilitation programs can be a source of self-care counseling, with a focus on education and skill development as well as an opportunity for frequent symptom assessment [31]. In 2014, the Centers for Medicare and Medicaid Services added cardiac rehabilitation services to beneficiaries with stable chronic HF [32]. However, at the time of this study, there was little opportunity for our study population to attend cardiac rehabilitation and most cited lack of insurance coverage. This finding is not new and highlights a health disparity for ethnic minority patients with HF. Racial disparities in the referral process to cardiac rehabilitation have been noted in the past. In a study of almost 2000 cardiac patients eligible for cardiac rehabilitation, Whites were more likely to be referred than Blacks (OR = 1.81; 95% CI: 1.22–2.68) even after controlling for age, education, socioeconomic status, and insurance [33]. The barriers to cardiac rehabilitation identified in our ethnic minority population sample, particularly lack of referral, were very similar to those in other populations. Our study adds to the literature by explicating the lack of exercise counseling, CR referral, and minimal awareness of the rehabilitation program in this population. It is important to note that many of these participants had non-ischemic HF that may have precluded providers from referring to CR given the lack of insurance coverage.

Our study reinforced that targeted education about the benefits of CR is needed, particularly in patients with low levels of education and low socioeconomic status who have had low adherence rates to CR in the past [34]. Education about medication adherence, maintenance of healthy body weight, and management of coexisting conditions (hypertension, diabetes) included in CR [29] may also benefit this population. In fact, the higher prevalence of HF in African Americans has been attributed to modifiable risk factors such as high blood pressure, high blood sugar and smoking. Obesity and physical inactivity are additional risk factors that may also be modified [35]. However, lack of a safe environment may inhibit engaging in physical activity in the surrounding neighborhood [35] and this systems level barrier is harder to providers to address. One solution may be to collaborate with local churches to promote a physical activity intervention, as the church is a known source of trust and would be an ideal partner between African Americans and the health care community [36]. A similar approach has been used in a comprehensive health counseling study set in a community health center. The aims of this study were to increase physical activity and improve the dietary habits in African American women at risk for heart disease [37].

Sociocultural influences have previously been reported elsewhere as an influence on overall self-care in this sample [15] and may also help to explain lower levels of physical activity in this population. Cogbill [38] explored whether sociocultural attitudes were associated with self-reported physical activity in African American adults age 45–75 (*n* = 446). Results indicate that individuals with strong concerns for family and community were more likely to report meeting recommended levels of physical activity. Our study adds to the current literature by exploring how this sample has adapted to living with the symptoms and limitations of HF. Some were still trying to do as much as possible, while others have given up trying to do what had been possible before they had HF. Participants spoke often of all that was lost: Playing ball with grandchildren; dancing at a party; swimming at a beach; shopping at a mall; or even going to church. Previous clinical trials indicate regular exercise is safe and can improve functional capacity in patients with HF [14]. The results of our study suggest a tailored approach that incorporates past physical activity preferences may be beneficial in promoting physical activity in this population.

In addition, physical activity in participants in our sample was associated with depressive symptoms. In accord with our finding of a high prevalence of depressive symptoms detected by the PHQ-9, a recent study using the Multiple Affective Adjective Checklist (MAACL) found that in patients diagnosed with HF, 63% had mean scores that exceeded the level for depression [39]. In subjects from the HF-ACTION (Heart Failure: A Controlled Trial Investigating Outcomes in Exercise Training) study, which was the largest randomized trial of exercise in HF, 28% had clinically significant levels of depressive symptoms [40].

Our results suggest that depression as a common comorbid condition plays a potent role in physical activity levels. Specifically, individuals with low exercise levels and PHQ-9 scores consistent with depressive symptoms described how mood influences ability to be physically active. Additionally, functional status was negatively correlated with depression; those with higher depression scores on PHQ-9 had lower functional status on the DASI. In a larger sample (*n* = 256; 18% minority) of heart failure patients, a change in depressive symptoms was the strongest predictor of one-year health-related quality of life, after controlling for functional status, demographics and other clinical factors [41]. In the HF-ACTION study, those subjects with a higher level of depression had a greater risk of HF hospitalization and HF death. But importantly, subjects randomized to the exercise program had significantly lower levels of depression as compared to the usual care group by three months, which persisted through the first year [40]. Given the triad of depression, low functional status, and poor health-related quality of life in patients with HF, identifying culturally appropriate physical activity interventions may provide improvements across these areas.

The patients in the current study had evidence of low functional capacity, but many were trying to stay active in their own way. Nevertheless, this low level of function may not preclude them from benefitting from regular physical activity. In the HF-ACTION study, subjects who identified as Black had lower baseline functional capacity compared to Whites as measured by the six-minute walk and cardiopulmonary exercise test [42]. Although they experienced higher HF hospitalization, there was no evidence Black subjects exhibited a differential response to the exercise training. The reason for this outcome is unclear, but still supports the use of routine exercise as therapy for all patients with HF.

Finally, our study was unique in its focus on physical activity as a component of self-care as guided by the situation specific model of self-care. Accordingly, self-care requires knowledge about and skill in the particular self-care behavior as well as compatibility with one’s values [16]. Our study revealed that the lack of information about engaging in physical activity or regular exercise was a barrier to participation. Sociocultural factors, including their desire to remain active with family and friends, also likely contributed to how individuals engaged in physical activity, both before and after their HF diagnosis. Culturally sensitive interventions that increase knowledge, and help individuals develop the necessary skills to safely engage in exercise, are needed.

### Limitations and Strengths

There were several limitations of this study, including the small sample size limited to African American participants, without other minority individuals. Although this sample size was appropriate for the qualitative aims, the quantitative analysis was limited. For example, the relationship between functional status, physical activity, and depression in an ethnic minority population needs to be fully explored in a larger sample. This study was cross-sectional and no causal links could be established between depression and physical activity. Additionally, the influence of family members’ or caregivers’ social support on levels of participants’ physical activity was not explored in this study. It has been found that with high levels of social support, patients with HF were more likely to exercise on a regular basis [43].

Health literacy is an important potential confounder that was not formally assessed in this study. The low education level of this sample was learned only during data collection. This may have affected their understanding of health information like physical activity instructions provided to them in the past. In addition, there may have been an element of social desirability in completing the instruments that may help explain lack of concordance in some cases [44]. Despite these limitations, our findings provide important new insight into the barriers that impact physical activity behavior in African Americans with HF.

## 5. Conclusions

Our findings highlight the need for the development of strategies to increase adherence to guideline recommendations for exercise in this population. This may be one avenue to reducing the disparate outcomes seen in African American with HF. Given the high prevalence of depression in this sample, additional work is required to better understand the interaction between depression, physical activity and HF. Additionally, understanding the influence of culture in minority patients with HF is essential in developing physical activity interventions. The perception of safety in engaging in physical activity for this vulnerable population may need further exploration. Providers may need to repeatedly endorse the benefits of physical activity for their patients. Finally, given the known benefits of CR on both functional status and depression, progress needs to be made in making this program not only affordable but accessible for all individuals with HF.
